# 238. Antimicrobial Activity of Dalbavancin against Gram-Positive Bacteria Isolated from Patients with Bone and Joint Infections from the United States (US) and Europe (2016-2020): Results from the International Dalbavancin Evaluation of Activity (IDEA) Program

**DOI:** 10.1093/ofid/ofab466.440

**Published:** 2021-12-04

**Authors:** Helio S Sader, Leonard R Duncan, Mariana Castanheira, Mariana Castanheira, Rodrigo E Mendes

**Affiliations:** JMI Laboratories, North Liberty, Iowa

## Abstract

**Background:**

Bone and joint infections (BJI) comprise a series of disorders, including septic arthritis, osteomyelitis, and prosthetic joint infections. Dalbavancin (DALBA) is a lipoglycopeptide with a very long half-life that allows the treatment of serious infections with once weekly or biweekly administration. We evaluated the activity of DALBA against pathogens isolated from BJI.

**Methods:**

A total of 798 organisms were collected from 62 US and 28 European (EU) hospitals in 2016-2020, including 503 *S. aureus*, 140 β-haemolytic streptococci (BHS), 71 coagulase-negative staphylococci (CoNS), 57 *Enterococcus* spp. (ESP), 22 viridans group streptococci (VGS), and 5 *S. pneumoniae*. Bacteria were identified by standard algorithms and MALDI-TOF-MS. Susceptibility testing was performed by the reference broth microdilution method in a central laboratory.

**Results:**

*S. aureus* (63.0%) was the most common pathogen associated with BJI, followed by BHS (17.5%), CoNS (8.9%), and ESP (7.1%). All *S. aureus* isolates were susceptible (S) to DALBA (MIC_50/90_, 0.03/0.03 mg/L), linezolid (LNZ; MIC_50/90_, 1/2 mg/L), teicoplanin (TEI; MIC_50/90_, ≤0.5/1 mg/L), vancomycin (VAN; MIC_50/90_, 1/1 mg/L), and daptomycin (DAPTO; MIC_50/90_, 0.25/0.0.5 mg/L. DALBA was 8- to 16-fold more potent than DAPTO and 32- to 64-fold more potent than LNZ, VAN, and TEI against *S. aureus*. Oxacillin resistance (OXA-R) rates among *S. aureus* (MRSA rates) were 35.5% and 15.4% in the US and EU, respectively. Ceftaroline (CPT) was active against 98.6% of *S. aureus* (MIC_50/90_, 0.25/1 mg/L) and 94.7% of MRSA (MIC_50/90_, 1/1 mg/L) isolates. Doxycycline and levofloxacin were active against 97.0% and 76.5% of *S. aureus*, respectively. Among CoNS, (54.9% OXA-R), DALBA (MIC_50/90_, 0.03/0.03 mg/L; highest MIC, 0.12 mg/L) was the most potent agent, followed by DAPTO (MIC_50/90_, 0.25/0.5 mg/L), CPT (MIC_50/90_, 0.25/0.5 mg/L) and LNZ (MIC_50/90_, 0.5/1 mg/L). The highest DALBA MIC value among BHS and VGS was 0.12 mg/L (MIC_90_, 0.03 mg/L for both groups). VAN was active against 82.4% of ESP and DALBA inhibited all VAN-S ESP at ≤0.06 mg/L.

**Conclusion:**

DALBA demonstrated potent *in vitro* activity against common gram-positive organisms (GP) causing BJI and appears to be a valuable option to treat BJI/osteomyelitis caused by GP.

**Disclosures:**

**Helio S. Sader, MD, PhD, FIDSA**, **AbbVie (formerly Allergan**) (Research Grant or Support)**Basilea Pharmaceutica International, Ltd.** (Research Grant or Support)**Cipla Therapeutics** (Research Grant or Support)**Cipla USA Inc.** (Research Grant or Support)**Department of Health and Human Services** (Research Grant or Support, Contract no. HHSO100201600002C)**Melinta Therapeutics, LLC** (Research Grant or Support)**Nabriva Therapeutics** (Research Grant or Support)**Pfizer, Inc.** (Research Grant or Support)**Shionogi** (Research Grant or Support)**Spero Therapeutics** (Research Grant or Support) **Leonard R. Duncan, PhD**, **AbbVie (formerly Allergan**) (Research Grant or Support)**Basilea Pharmaceutica International, Ltd.** (Research Grant or Support)**Cipla Therapeutics** (Research Grant or Support)**Cipla USA Inc.** (Research Grant or Support)**Department of Health and Human Services** (Research Grant or Support, Contract no. HHSO100201600002C)**Shionogi** (Research Grant or Support) **Mariana Castanheira, PhD**, **AbbVie (formerly Allergan**) (Research Grant or Support)**Bravos Biosciences** (Research Grant or Support)**Cidara Therapeutics, Inc.** (Research Grant or Support)**Cipla Therapeutics** (Research Grant or Support)**Cipla USA Inc.** (Research Grant or Support)**GlaxoSmithKline** (Research Grant or Support)**Melinta Therapeutics, Inc.** (Research Grant or Support)**Melinta Therapeutics, LLC** (Research Grant or Support)**Pfizer, Inc.** (Research Grant or Support)**Qpex Biopharma** (Research Grant or Support)**Shionogi** (Research Grant or Support)**Spero Therapeutics** (Research Grant or Support) **Mariana Castanheira, PhD**, Affinity Biosensors (Individual(s) Involved: Self): Research Grant or Support; Allergan (Individual(s) Involved: Self): Research Grant or Support; Amicrobe, Inc (Individual(s) Involved: Self): Research Grant or Support; Amplyx Pharma (Individual(s) Involved: Self): Research Grant or Support; Artugen Therapeutics USA, Inc. (Individual(s) Involved: Self): Research Grant or Support; Astellas (Individual(s) Involved: Self): Research Grant or Support; Basilea (Individual(s) Involved: Self): Research Grant or Support; Beth Israel Deaconess Medical Center (Individual(s) Involved: Self): Research Grant or Support; BIDMC (Individual(s) Involved: Self): Research Grant or Support; bioMerieux Inc. (Individual(s) Involved: Self): Research Grant or Support; BioVersys Ag (Individual(s) Involved: Self): Research Grant or Support; Bugworks (Individual(s) Involved: Self): Research Grant or Support; Cidara (Individual(s) Involved: Self): Research Grant or Support; Cipla (Individual(s) Involved: Self): Research Grant or Support; Contrafect (Individual(s) Involved: Self): Research Grant or Support; Cormedix (Individual(s) Involved: Self): Research Grant or Support; Crestone, Inc. (Individual(s) Involved: Self): Research Grant or Support; Curza (Individual(s) Involved: Self): Research Grant or Support; CXC7 (Individual(s) Involved: Self): Research Grant or Support; Entasis (Individual(s) Involved: Self): Research Grant or Support; Fedora Pharmaceutical (Individual(s) Involved: Self): Research Grant or Support; Fimbrion Therapeutics (Individual(s) Involved: Self): Research Grant or Support; Fox Chase (Individual(s) Involved: Self): Research Grant or Support; GlaxoSmithKline (Individual(s) Involved: Self): Research Grant or Support; Guardian Therapeutics (Individual(s) Involved: Self): Research Grant or Support; Hardy Diagnostics (Individual(s) Involved: Self): Research Grant or Support; IHMA (Individual(s) Involved: Self): Research Grant or Support; Janssen Research & Development (Individual(s) Involved: Self): Research Grant or Support; Johnson & Johnson (Individual(s) Involved: Self): Research Grant or Support; Kaleido Biosceinces (Individual(s) Involved: Self): Research Grant or Support; KBP Biosciences (Individual(s) Involved: Self): Research Grant or Support; Luminex (Individual(s) Involved: Self): Research Grant or Support; Matrivax (Individual(s) Involved: Self): Research Grant or Support; Mayo Clinic (Individual(s) Involved: Self): Research Grant or Support; Medpace (Individual(s) Involved: Self): Research Grant or Support; Meiji Seika Pharma Co., Ltd. (Individual(s) Involved: Self): Research Grant or Support; Melinta (Individual(s) Involved: Self): Research Grant or Support; Menarini (Individual(s) Involved: Self): Research Grant or Support; Merck (Individual(s) Involved: Self): Research Grant or Support; Meridian Bioscience Inc. (Individual(s) Involved: Self): Research Grant or Support; Micromyx (Individual(s) Involved: Self): Research Grant or Support; MicuRx (Individual(s) Involved: Self): Research Grant or Support; N8 Medical (Individual(s) Involved: Self): Research Grant or Support; Nabriva (Individual(s) Involved: Self): Research Grant or Support; National Institutes of Health (Individual(s) Involved: Self): Research Grant or Support; National University of Singapore (Individual(s) Involved: Self): Research Grant or Support; North Bristol NHS Trust (Individual(s) Involved: Self): Research Grant or Support; Novome Biotechnologies (Individual(s) Involved: Self): Research Grant or Support; Paratek (Individual(s) Involved: Self): Research Grant or Support; Pfizer (Individual(s) Involved: Self): Research Grant or Support; Prokaryotics Inc. (Individual(s) Involved: Self): Research Grant or Support; QPEX Biopharma (Individual(s) Involved: Self): Research Grant or Support; Rhode Island Hospital (Individual(s) Involved: Self): Research Grant or Support; RIHML (Individual(s) Involved: Self): Research Grant or Support; Roche (Individual(s) Involved: Self): Research Grant or Support; Roivant (Individual(s) Involved: Self): Research Grant or Support; Salvat (Individual(s) Involved: Self): Research Grant or Support; Scynexis (Individual(s) Involved: Self): Research Grant or Support; SeLux Diagnostics (Individual(s) Involved: Self): Research Grant or Support; Shionogi (Individual(s) Involved: Self): Research Grant or Support; Specific Diagnostics (Individual(s) Involved: Self): Research Grant or Support; Spero (Individual(s) Involved: Self): Research Grant or Support; SuperTrans Medical LT (Individual(s) Involved: Self): Research Grant or Support; T2 Biosystems (Individual(s) Involved: Self): Research Grant or Support; The University of Queensland (Individual(s) Involved: Self): Research Grant or Support; Thermo Fisher Scientific (Individual(s) Involved: Self): Research Grant or Support; Tufts Medical Center (Individual(s) Involved: Self): Research Grant or Support; Universite de Sherbrooke (Individual(s) Involved: Self): Research Grant or Support; University of Iowa (Individual(s) Involved: Self): Research Grant or Support; University of Iowa Hospitals and Clinics (Individual(s) Involved: Self): Research Grant or Support; University of Wisconsin (Individual(s) Involved: Self): Research Grant or Support; UNT System College of Pharmacy (Individual(s) Involved: Self): Research Grant or Support; URMC (Individual(s) Involved: Self): Research Grant or Support; UT Southwestern (Individual(s) Involved: Self): Research Grant or Support; VenatoRx (Individual(s) Involved: Self): Research Grant or Support; Viosera Therapeutics (Individual(s) Involved: Self): Research Grant or Support; Wayne State University (Individual(s) Involved: Self): Research Grant or Support **Rodrigo E. Mendes, PhD**, **AbbVie** (Research Grant or Support)**AbbVie (formerly Allergan**) (Research Grant or Support)**Cipla Therapeutics** (Research Grant or Support)**Cipla USA Inc.** (Research Grant or Support)**ContraFect Corporation** (Research Grant or Support)**GlaxoSmithKline, LLC** (Research Grant or Support)**Melinta Therapeutics, Inc.** (Research Grant or Support)**Melinta Therapeutics, LLC** (Research Grant or Support)**Nabriva Therapeutics** (Research Grant or Support)**Pfizer, Inc.** (Research Grant or Support)**Shionogi** (Research Grant or Support)**Spero Therapeutics** (Research Grant or Support)

